# Micro-Droplet Platform for Exploring the Mechanism of Mixed Field Agglutination in B_3_ Subtype

**DOI:** 10.3390/bios11080276

**Published:** 2021-08-16

**Authors:** Ding-Ping Chen, Chen Chen, Pei-Yu Wu, Yen-Heng Lin, Wei-Tzu Lin, Yi-Liang Yan

**Affiliations:** 1 Department of Laboratory Medicine, Chang Gung Memorial Hospital, Taoyuan City 333, Taiwan; a12048@cgmh.org.tw (D.-P.C.); berry0908@cgmh.org.tw (W.-T.L.); alexttt@cgmh.org.tw (Y.-L.Y.); 2 Department of Medical Biotechnology and Laboratory Science, College of Medicine, Chang Gung University, Taoyuan City 333, Taiwan; 3 Graduate Institute of Biomedical Sciences, College of Medicine, Chang Gung University, Taoyuan City 333, Taiwan; 4 Department of Electronic Engineering, Chang Gung University, Taoyuan City 333, Taiwan; 0521janechen@gmail.com (C.C.); rofer162@gmail.com (P.-Y.W.); 5 Graduate Institute of Biomedical Engineering, Chang Gung University, Taoyuan City 333, Taiwan; 6 Department of Otolaryngology-Head and Neck Surgery, Chang Gung Memorial Hospital, Taoyuan City 333, Taiwan

**Keywords:** B_3_ subtyping, microfluidics, blood agglutination

## Abstract

B_3_ is the most common subtype of blood group B in the Taiwanese population, and most of the B_3_ individuals in the Taiwanese population have the IVS3 + 5 G > A (rs55852701) gene variation. Additionally, a typical mixed field agglutination is observed when the B_3_ subtype is tested with anti-B antibody or anti-AB antibody. The molecular biology of the gene variation in the B_3_ subtype has been identified, however, the mechanism of the mixed field agglutination caused by the type B_3_ blood samples is still unclear. Therefore, the purpose of this study was to understand the reason for the mixed field agglutination caused by B_3_. A micro-droplet platform was used to observe the agglutination of type B and type B_3_ blood samples in different blood sample concentrations, antibody concentrations, and at reaction times. We found that the agglutination reaction in every droplet slowed down with an increase in the dilution ratio of blood sample and antibody, whether type B blood or type B_3_ blood was used. However, as the reaction time increased, the complete agglutination in the droplet was seen in type B blood, while the mixed field agglutination still occurred in B_3_ within 1 min. In addition, the degree of agglutination was similar in each droplet, which showed high reproducibility. As a result, we inferred that there are two types of cells in the B_3_ subtype that simultaneously create a mixed field agglutination, rather than each red blood cell carrying a small amount of antigen, resulting in less agglutination.

## 1. Introduction

B_3_ is the most common subtype of blood group B. From genetic analysis studies, it was found that most of the B_3_ patients in the Taiwanese population have the IVS3 + 5 G > A (rs55852701) gene mutation, which causes splicing errors and prevents generation of the part of the functional protein encoded by exon 3 [1,2]. It is worth noting that when the B_3_ subtype is tested with anti-B antibody or anti-AB antibody for forward typing, a typical mixed field agglutination is observed. The phenomenon of mixed field agglutination may occur when the specimen is weak A phenotype, weak B phenotype, or weak RhD phenotype, or the loss of red blood cell antigen is caused by hematopoietic malignant tumors [3], and it may also occur when there are two or more types of cells in a specimen, such as the chimeric state of embryo fusion, twin hematopoiesis, and stem cell transplantation [4,5,6]. However, not all the weak phenotypes have mixed field agglutination when forward typing. For example, B_el_ is one of weak phenotypes in blood group B not generating mixed field agglutination with the anti-B antibody [7]. Furthermore, A_3_ is the only weak phenotype of blood group A that generates a mixed field agglutination with anti-A antibody, excepting other A subtypes [8]. In addition, there are many kinds of gene variations in ABO subtypes, including substitution, splice-site mutation, insertion, and deletion, that have different effects on the subsequent phenotypes [9]. We identified the effects of IVS3 + 5 G > A (rs55852701) on B_3_ in a previous study [2], however, the mechanism of the mixed field agglutination caused by the type B_3_ blood samples from the patients without embryo fusion or stem cell transplantation remains unclear. Therefore, the purpose of this study was to understand the reason for mixed field agglutination caused by B_3_.

Microfluidic technology has been widely used in biological applications, including disease diagnostics [10], DNA [11] and protein [12] analysis, and cell study [13]. Because of its small channel dimension size, it is suitable for analysis of tiny volume samples, such as neonates’ blood, and the reaction time is accelerated in a small reaction space. For example, a digital microfluidic platform was adopted for a newborn screening laboratory. A submicroliter sample was used for immunoassays, enzyme assays, and DNA-based assays. The system reduced the operation time compared with the current benchtop [14]. In addition, a micro glass capillary-based platform was proposed to demonstrate the enzyme-linked immunosorbent assay (ELISA). The capillary-based ELISA platform reduced the sample volume to 20 μL and shortened the assay time to 16 min, which is a 10-fold and 5-fold reduction in assay time and sample volume, respectively, compared to the traditional plate-based method [15]. Among these microfluidic platforms, the droplet-based microfluidic platform is a valuable tool for various applications, such as single-cell analysis [16], digital polymerase chain reaction (PCR) [17], biological assays [18], and drug screening [19]. Droplets can be generated at kHz frequencies and the sample/reagent can be compartmentalized into pico- or nanoliter droplets, which enables the high-throughput or high-reproducibility analysis [20]. In addition, droplets can be manipulated in a variety of ways to perform versatile applications, such as droplet split [21] and droplet merging [22]. In droplet merging, a reagent in one droplet can be injected into another droplet to control the starting time of the reaction [23]. Droplets can also be sorted by the dielectrophoretic force [24], and cells [25] or bacteria [26] can be incubated in a droplet for up to 1 h. In addition, microfluidics has gained much interest for blood typing applications. For example, S. Karimi proposed a microfluidic chip with a blood–plasma separator that can simultaneously perform both direct and indirect blood typing [27]. The blood sample is self-driven and does not need any external forces. In addition, a finger actuation microfluidic chip was proposed [28]. Pushing only seven buttons can achieve blood typing. A cyclo-olefin polymer-based microfluidic chip was proposed for subtyping blood groups and detecting anemia and polycythemia vera [29]. By means of blood agglutination with different channel lengths, users can determine whether they are sick or have a rare blood type. Furthermore, a paper-based device can also be used for fast blood typing, and the cost is relatively lower than the cost of other devices [30].

Among these microfluidic techniques, the micro-droplet platform was used to observe differences in red blood cell agglutination when the forward typing of blood type B and its subtype, type B_3_, was carried out. The use of the micro-droplet platform decreases the required volume of red blood cell samples and antibodies, which is especially important for rare blood types or neonatal blood samples. It also confines the blood sample to a tiny space, which increases the collision possibility of the antigen and antibody and thus accelerates the reaction. The micro droplets properly partition the sample as many identical instances, which avoids the nonuniform agglutination of a tube or microwell and increases the accuracy of the agglutination study or detection.

## 2. Materials and Methods

### 2.1. Microfluidic Chip Design and Fabrication

**Figure 1:**
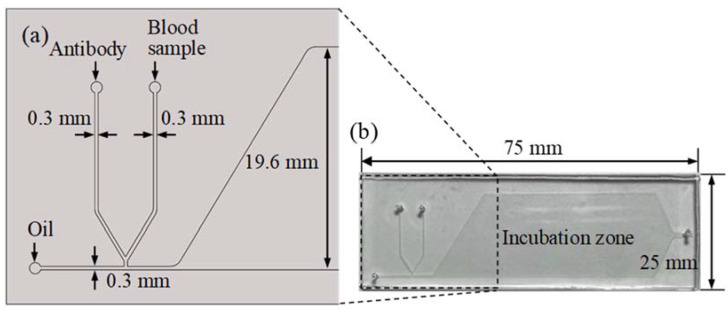
(**a**) The schematic illustration of the chip design. The microchannel’s width and height are 0.3 mm and 0.1 mm, respectively. (**b**) Photograph of the chip. The size of the microfluidic chip is 75 × 25 mm.

The micro-droplet-based blood typing chip was fabricated with a standard soft lithography process composed of two layers. The top layer was a PDMS layer with a microchannel and an incubation chamber. The bottom layer was a glass substrate. The glass slide was spin-coated with a thin PDMS to ensure that all the microchannels had the same surface property. The microchannel was treated with Aquapel (PPG Industries, Pittsburgh, PA, USA) to form a hydrophobic property before the sample and oil were injected [31]. The schematic illustration of the chip design is shown in Figure 1a. A T-junction microchannel was used for the droplet formation. Two side channels with 300 μm widths were merged near the T-junction. Packed red blood cells from the donor and anti-B antibodies were injected into the two side channels and merged as a water phase. The water phase was then cut by the oil phase to form droplets. The droplets flowed into an incubation chamber for the red blood cell agglutination reaction. The microchannel and incubator chamber were 100 μm in height. The size of the microfluidic chip was 25 × 75 mm, as shown in Figure 1b.

### 2.2. Experimental Set-Up

**Figure 2:**
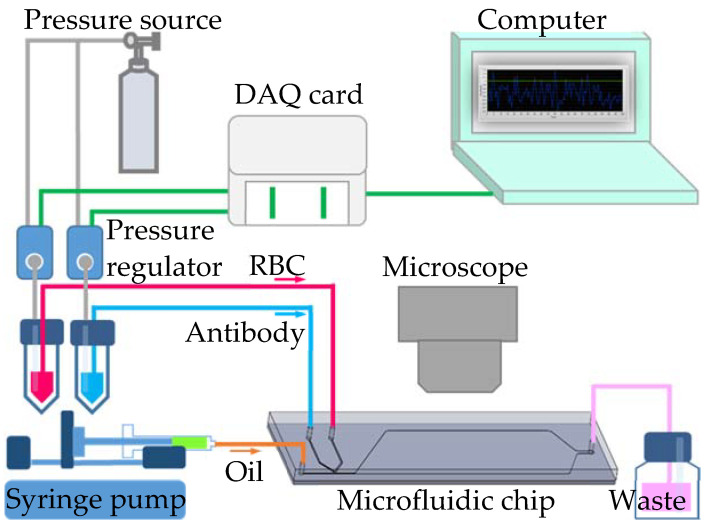
Schematic illustration of the experimental set-up.

To generate uniform-sized droplets for the red blood cell agglutination reaction, both side channel flow rates were controlled by a pressure-driven flow with pressure regulators (VSO-EP-Miniature Pressure Controller, Parker, NA, USA). The pressure regulators were precisely controlled by LabVIEW software (Version 11.0, National Instruments, Austin, TX, USA) with an interface card (DAQ, USB-6281, National Instruments). The continuous phase with fluorinate oil (HFE-7500, 3M, Saint Paul, MN, USA) was introduced by a syringe pump (Legato 180, KD Scientific Inc., Holliston, MA, USA). Red blood cell agglutination reaction in the droplets was observed under a microscope (BX43, Olympus, Tokyo, Japan) and images were recorded with a digital camera (ILCE-5100Y, Sony, Tokyo, Japan). The presence of agglutination was quantitatively evaluated by monitoring a decrease in the area ratio of the blood cell image in the droplet over time. The area ratio was analyzed using ImageJ software. The schematic illustration of the experimental set-up is found in Figure 2.

### 2.3. Emulsification of Agglutination Reactions

The aqueous solutions with packed red blood cells (10 μL) and the anti-B antibodies (10 μL) were placed in 200 μL microcentrifuge tubes. The blood sample and antibody solutions were then driven into the two side channels at an air pressure of 5.6 kPa through the same length of polytetrafluoroethylene (PTFE) tubing (0.15 mm i.d., 1.59 mm o.d.). Fluorinated oil containing 2% (*w*/*w*) surfactant (a PEG–PFPE amphiphilic block copolymer) was placed in a 10 mL syringe and introduced with a fixed flow rate of 120 μL/min. When the shear force provided by the oil phase was higher than the interfacial force in the aqueous phase, the breakup of dispersed droplets was generated to form reaction incubators with a production frequency of approximately 100 Hz and a diameter of approximately 300 μm. Equal volumes of blood sample and antibody were encapsulated in the droplets. Thus, within several seconds, hundreds of agglutination reactions occurred simultaneously in each droplet.

### 2.4. Sample and Reagent Preparation

The anti-B antibody was purchased from IMMUCOR (Murine Monoclonal, IMMUCOR, Peachtree Corners, GA, USA). The human blood specimens were collected with the consent of the volunteers, and the study was approved by the Institutional Review Board of Chang Gung Memorial Hospital Medical Center (IRB number: 201800548B0C501). All methods were performed in accordance with the relevant guidelines and regulations. Informed consent was obtained from all volunteers. Based on their specific gravity, the blood components were distinguished by low-speed centrifuge at 1700× *g* (1000 rpm) for 20 min. After centrifugation, the packed red blood cells were in the lowest layer. Packed red blood cells and anti-B antibodies were serially diluted with normal saline solution (sterile non-pyrogenic).

## 3. Results

**Figure 3:**
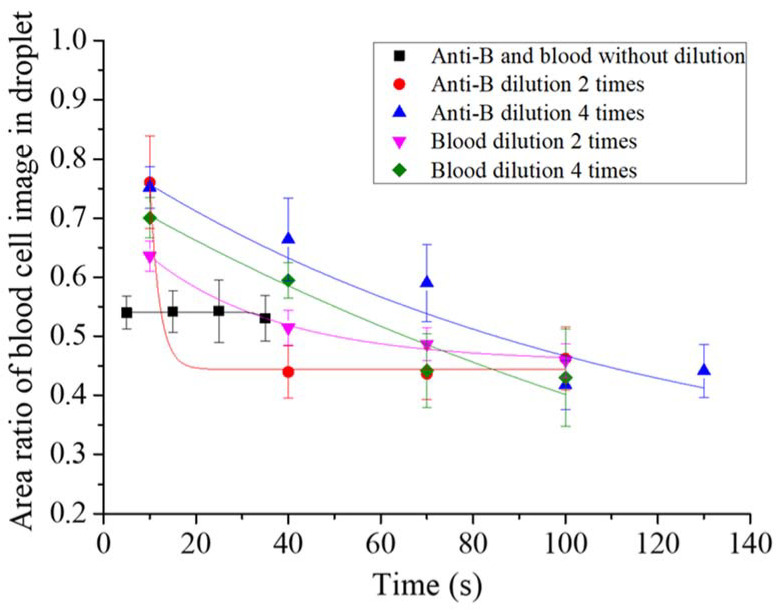
Quantitative analysis of the red blood cell agglutination extent in droplets from different experimental groups. The area ratio of the blood cell image in the droplet was used to evaluate the presence of agglutination.

First, we tested the optimal reaction concentrations of the blood sample and antibody solutions in the micro-droplet platform. The undiluted blood sample was reacted with the original concentration of the two-times-diluted anti-B antibody and the four-times-diluted antibody samples. Following that, the undiluted anti-B antibody sample was reacted with the original concentration of blood sample, the two-times-diluted and the four-times-diluted blood samples. The agglutination extent was described quantitatively for the type B blood reaction. Each experimental group reaction was recorded, and we evaluated the presence of agglutination by monitoring a decrease in the area ratio of the blood cell image in the droplet over time. At the beginning of the reaction, red blood cells were distributed uniformly in the droplets, thus the area ratio of the blood cell image was large. The ratio decreased over time when more and more cell agglutination took place, as shown in Figure 3. We observed that the agglutination in the experimental group without blood dilution or antibody immediately reached (within 3 s) a steady state when they encapsulated in the droplet with an area ratio of 0.53–0.54. The agglutination rate was slowest with the experimental group of four-times-diluted antibody. The area ratio of the blood cell image in the droplet dropped from 0.75 to 0.44 over 130 s.

Table 1 shows all the experiment results. The detailed image of the type B blood agglutination reaction is shown in Supplementary Video S1. As reaction time increased, agglutination also occurred for the non-agglutinated red blood cells after they collided with the main agglutinated clump. As the dilution factor of the anti-B antibody concentration or the blood sample concentration increased, the ratio of red blood cells that immediately agglutinated after droplet formation decreased. From this, we saw that as the number of antibodies and red blood cells decreased, the probability of collision between them decreased, which decreased the agglutination reaction rate. In addition, the number of antibodies in the original concentration, the two-times dilution, and the four-times dilution were sufficient for complete agglutination of the red blood cells. Furthermore, type B_3_ blood samples were also studied using the aforementioned testing conditions. Note that the extent of the mixed field agglutination formed by the type B_3_ blood reaction was difficult to analyze quantitatively using images. Steady state agglutination times of different experimental groups were evaluated with videos. The experimental results showed that the reaction between the undiluted blood and the undiluted antibody samples was the fastest. However, the ratio of agglutinated red blood cells at the instant of droplet formation was significantly lower than that for type B blood. The detailed image of the type B_3_ blood agglutination reaction is shown in Supplementary Video S2. Furthermore, as the anti-B antibody concentration or blood sample concentration decreased due to dilution, the time for blood cell agglutination to reach a steady state increased. In the dilution experiments, the agglutination time and ratio of the agglutinated red blood cells when type B and type B_3_ blood reacted with antibodies in the microdroplets were significantly different. It is noteworthy that complete agglutination did not occur for the type B_3_ blood sample regardless of dilution conditions, in contrast to the type B blood sample. Therefore, we observed for an extended time the reaction of the undiluted blood sample and the undiluted antibody sample in the droplets to determine whether complete agglutination did not indeed occur in the type B_3_ blood sample or whether this was due to insufficient reaction time.

**Figure 4:**
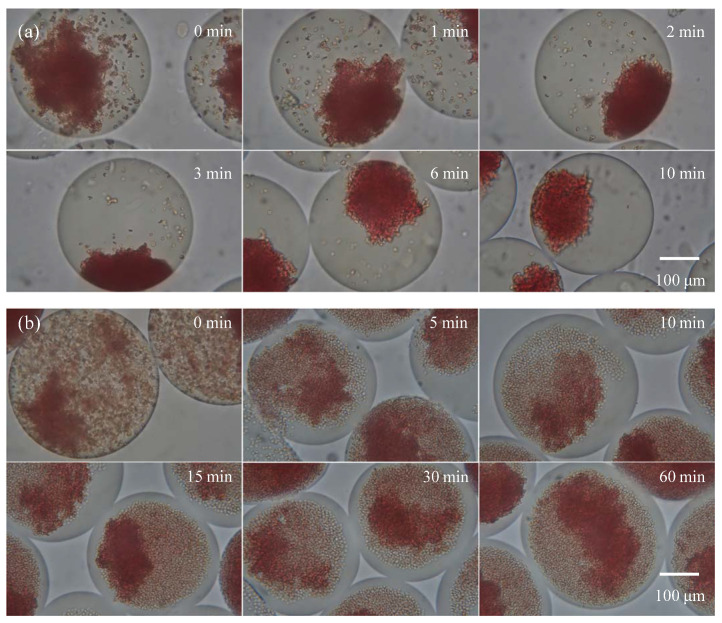
The agglutination of (**a**) type B and (**b**) subtype B_3_ blood at each time point. The sample and antibody concentrations were not diluted.

For this experiment, the undiluted type B and type B_3_ blood samples were encapsulated with the undiluted anti-B antibody sample in droplets for agglutination. A 20X objective lens was used for observation. Figure 4a shows the agglutination reaction for type B blood: When droplets were formed, steady state agglutination was seen immediately (within 3 s). Figure 4b shows the agglutination reaction for type B_3_ blood with an extended agglutination time reaction. After the droplet was formed, immediate agglutination occurred, which was similar to the results for type B blood. However, the ratio of agglutinated red blood cells was lower than type B blood, and steady state was reached at more than 120 s of reaction, i.e., the ratio of agglutinated red blood cells no longer increased. At this point, red blood cells settled to the bottom of the drop from gravity. Therefore, most red blood cells were within the depth of field, allowing for clearer observation of the agglutination. When the reaction time increased to 60 min, there was no further change in the ratio of agglutinated red blood cells in the droplet, and a similar ratio of agglutinated red blood cells was present in every drop. Note that the observation areas in Figure 4 are fixed. Because the observation time lasted an hour and the droplets moved slightly in the micro chamber, different droplets were captured in the video clips. The variation between different droplet images was less because of the highly consistent reaction in the droplet platform, which is crucial for the study of the mechanism of blood agglutination.

## 4. Discussion

In our previous study [2], we found that the splicing caused by the IVS3 + 5 G > A mutation was very complicated and might produce a variety of different splicing transcripts, but only the splicing transcript that removed exon 3 alone could produce functional B glycosyltransferase. The nucleotide elimination of exon 3 skipping was an exact multiple of 3, resulting in the loss of 19 amino acids at the N-terminal segment rather than the frame shift. However, its proportion was only 1%, and the remaining 99% of splicing transcripts could not produce functional B glycosyltransferase, so the B glycosyltransferase activity was reduced and resulted in a weak B phenotype. After that, the cell model constructed of K562 cells was used to exclude the influence of the regulation of promoter and enhancer on the ABO gene. According to the previous study, we confirmed that the weak expression of the B antigen on B_3_ and the production of a mixed field were caused by the exon 3 deletion resulting from the IVS3 + 5 G > A mutation, which led to the production of truncated but functional B glycosyltransferase. It also proved that the individual B_3_ protein was sufficient to cause mixed field agglutination. Moreover, that research proposed two inter-related mechanisms for the weak expression of the B antigen and mixed field agglutination in the B_3_ phenotype. Firstly, only about 1% of splicing transcripts caused by the IVS3 + 5 G > A mutation could produce functional B glycosyltransferase. Additionally, the IVS3 + 5 G > A mutation resulted in a failure to generate the transmembrane and stem region on B glycosyltransferases corresponding to the exon 3 sequence. This reduced the flexibility of the stem region, and it could not transfer 1,3-d-galactose to the H protein effectively.

In the present study, agglutination reactions of both type B and type B_3_ blood slowed down when the dilution factor of the blood sample and antibody was increased. As reaction time increased, complete agglutination in the droplet occurred in type B blood. It was inferred that every red blood cell in type B blood carried the abundant antigens. In contrast, complete agglutination was not achieved for type B_3_ blood in the droplets. The ratio of agglutinated red blood cells was similar in each droplet. We supposed that there were two possible reasons for the slow agglutination reaction of type B_3_ blood. Firstly, every red blood cell in type B_3_ blood carries fewer antigens, which decreases agglutination force. Secondly, a certain proportion of red blood cells in type B_3_ blood may not carry the antigen. When the undiluted type B_3_ blood and undiluted antibody samples reacted for a long period of time, the sample still could not reach complete agglutination. Therefore, we speculated that a percentage of the red blood cells in type B_3_ blood may not carry the antigen. In addition to experimental evidence supporting the claim in the conclusion, we noted that clinical phenomena supported our conclusion. Red blood cell antigen changes can be seen in clinical cases of allogeneic hematopoietic stem cell transplantation [32]. For example, when a patient with type B blood receives type O blood from a donor with transplanted hematopoietic stem cells, the blood type gradually changes from type B to type B_3_, and even to type O, which was evaluated by forward typing and reverse typing. Moreover, mixed field agglutination may also be observed in blood transfusion, fetomaternal hemorrhage, and twin or dispermic chimerism [33], meaning two types of blood are mixed. Therefore, it is possible for type B_3_ blood to have B blood cells and O blood cells at the same time.

## 5. Conclusions

Combining the results of our previous study and this study, we found that the cause of mixed field agglutination in the B_3_ subtype was two types of cells in the B_3_ subtype simultaneously creating a mixed field agglutination, rather than each red blood cell carrying a small amount of antigen, resulting in less agglutination. In addition, the undiluted blood and undiluted antibody samples can be used to rapidly distinguish between the two blood types within 1 min. In clinical practice, laborious operations and errors can also be avoided by neglecting the dilution process.

## Figures and Tables

**Table 1:** Anti-B antibody sample and blood sample dilution effects on the agglutination of B and B_3_ blood types.

Anti-B Antibody Dilution (Times)	0	2	4	0	0
Blood sample dilution (times)	0	0	0	2	4
Type B blood agglutination	4+ (Video S1)	4+	4+	4+	4+
Time to reach steady state agglutination	<3 s	<40 s	<130 s	<40 s	<70 s
Type B_3_ blood agglutination	(Video S2)	Mixed field agglutination			
Time to reach steady state agglutination	>120 s	>200 s	>180 s	>210 s	>250 s

## Data Availability

Not applicable.

## Supplementary Materials

The following are available online at https://www.mdpi.com/article/10.3390/bios11080276/s1, Video S1: The reaction between the undiluted type B blood and undiluted antibody samples in the micro-droplet platform, Video S2: The reaction between the undiluted type B_3_ blood and undiluted antibody samples in the micro-droplet platform.

## References

[1] Yu L.-C., Twu Y.-C., Chou M.-L., Chang C.-Y., Wu C.-Y., Lin M. (2002). Molecular genetic analysis for the B3 allele. Blood.

[2] Chen D.-P., Tseng C.-P., Wang W.-T., Sun C.-F. (2012). Genetic and Mechanistic Evaluation for the Mixed-Field Agglutination in B3 Blood Type with IVS3+5G>A ABO Gene Mutation. PLoS ONE.

[3] Sheppard C.A., O’Reilly K.C., Schniederjan S.D., Hillyer C.D., Roback J.D. (2006). Detection of mixed-field agglutination due to loss of red cell antigen in hematopoietic malignancy. Transfusion.

[4] Cho D., Lee J.S., Yazer M.H., Song J.W., Shin M.G., Shin J.H., Suh S.P., Jeon M.J., Kim J.Y., Park J.T. (2006). Chimerism and mosaicism are important causes of ABO phenotype and genotype discrepancies. Immunohematology.

[5] Bluth M.H., Reid M.E., Manny N. (2007). Chimerism in the Immunohematology Laboratory in the Molecular Biology Era. Transfus. Med. Rev..

[6] Sharpe C., Lane D., Cote J., Hosseini-Maaf B., Goldman M., Olsson M.L., Hult A.K. (2014). Mixed field reactions in ABO and Rh typing chimerism likely resulting from twin haematopoiesis. Blood Transfus..

[7] Salmon C. (1976). Les phénotypes B faibles B3, Bx, Bel Classification pratique proposée. Rev. Française Transfus. Immuno-Hématologie.

[8] Armstrong B., Hardwick J., Raman L., Smart E., Wilkinson R. (2008). Introduction to Blood Transfusion Technology. ISBT Sci. Ser..

[9] Ying Y., Hong X., Xu X., Chen S., He J., Zhu F., Xie X. (2020). Molecular Basis of ABO Variants Including Identification of 16 Novel ABO Subgroup Alleles in Chinese Han Population. Transfus. Med. Hemotherapy.

[10] Ghosh S., Aggarwal K., Upaassana V.T., Nguyen T., Han J., Ahn C.H. (2020). A new microchannel capillary flow assay (MCFA) platform with lyophilized chemiluminescence reagents for a smartphone-based POCT detecting malaria. Microsyst. Nanoeng..

[11] Furlan C., Dirks R.A.M., Thomas P.C., Jones R.C., Wang J., Lynch M., Marks H., Vermeulen M. (2019). Miniaturised interaction proteomics on a microfluidic platform with ultra-low input requirements. Nat. Commun..

[12] Kane K.I.W., Moreno E.L., Hachi S., Walter M., Jarazo J., Oliveira M.A.P., Hankemeier T., Vulto P., Schwamborn J.C., Thoma M. (2019). Automated microfluidic cell culture of stem cell derived dopaminergic neurons. Sci. Rep..

[13] Tan X., Khaing Oo M.K., Gong Y., Li Y., Zhu H., Fan X. (2017). Glass capillary based microfluidic ELISA for rapid diagnostics. Analyst.

[14] Doufène K., Tourné-Péteilh C., Etienne P., Aubert-Pouëssel A. (2019). Microfluidic Systems for Droplet Generation in Aqueous Continuous Phases: A Focus Review. Langmuir.

[15] Agnihotri S.N., Raveshi M.R., Bhardwaj R., Neild A. (2020). Microfluidic Valves for Selective on-Chip Droplet Splitting at Multiple Sites. Langmuir.

[16] Zhang H., Guzman A.R., Wippold J.A., Li Y., Dai J., Huang C., Han A. (2020). An ultra high-efficiency droplet microfluidics platform using automatically synchronized droplet pairing and merging. Lab Chip.

[17] Sesen M., Whyte G. (2020). Image-Based Single Cell Sorting Automation in Droplet Microfluidics. Sci. Rep..

[18] Kulkarni M.B., Goel S. (2020). Advances in continuous-flow based microfluidic PCR devices—A review. Eng. Res. Express.

[19] Li J., Kim C.-J.C. (2020). Current commercialization status of electrowetting-on-dielectric (EWOD) digital microfluidics. Lab Chip.

[20] Matuła K., Rivello F., Huck W.T.S. (2020). Single-Cell Analysis Using Droplet Microfluidics. Adv. Biosyst..

[21] Falzone L., Musso N., Gattuso G., Bongiorno D., Palermo C.I., Scalia G., Libra M., Stefani S. (2020). Sensitivity assessment of droplet digital PCR for SARS-CoV-2 detection. Int. J. Mol. Med..

[22] O’Hara R., Tedone E., Ludlow A., Huang E., Arosio B., Mari D., Shay J.W. (2019). Quantitative mitochondrial DNA copy number determination using droplet digital PCR with single-cell resolution. Genome Res..

[23] Kulesa A., Kehe J., Hurtado J.E., Tawde P., Blainey P.C. (2018). Combinatorial drug discovery in nanoliter droplets. Proc. Natl. Acad. Sci. USA.

[24] Holland-Moritz D.A., Wismer M.K., Mann B.F., Farasat I., Devine P., Guetschow E.D., Mangion I., Welch C.J., Moore J.C., Sun S. (2020). Mass Activated Droplet Sorting (MADS) Enables High-Throughput Screening of Enzymatic Reactions at Nanoliter Scale. Angew. Chem. Int. Ed..

[25] Gérard A., Woolfe A., Mottet G., Reichen M., Castrillon C., Menrath V., Ellouze S., Poitou A., Doineau R., Briseno-Roa L. (2020). High-throughput single-cell activity-based screening and sequencing of antibodies using droplet microfluidics. Nat. Biotechnol..

[26] Karamitros C.S., Morvan M., Vigne A., Lim J., Gruner P., Beneyton T., Vrignon J., Baret J.-C. (2020). Bacterial Expression Systems for Enzymatic Activity in Droplet-Based Microfluidics. Anal. Chem..

[27] Karimi S., Mehrdel P., Farré-Lladós J., Casals-Terré J. (2019). A passive portable microfluidic blood–plasma separator for simultaneous determination of direct and indirect ABO/Rh blood typing. Lab Chip.

[28] Park J., Park J.-K. (2019). Finger-Actuated Microfluidic Display for Smart Blood Typing. Anal. Chem..

[29] Lin J.-H., Tsai T.-T., Zeng Q., Chang C.-Y., Guo J.-Y., Lin C.-J., Chen C.-F. (2020). A Multifunctional Microfluidic Device for Blood Typing and Primary Screening of Blood Diseases. ACS Sens..

[30] Hertaeg M.J., Tabor R.F., McLiesh H., Garnier G. (2021). A rapid paper-based blood typing method from droplet wicking. Analyst.

[31] Rotem A., Abate A.R., Utada A.S., Van Steijn V., Weitz D.A. (2012). Drop formation in non-planar microfluidic devices. Lab Chip.

[32] Yu Z.-Q., Gao Z.-F., Li H.-Y. (2012). Study on serological blood group conversion rule and clinical blood transfusion in allogeneic hematopoietic stem cell transplantation. Zhonghua Xue Ye Xue Za Zhi.

[33] Roback J.D., Grossman B.J., Harris T., Hillyer C.D. (2011). Technical Manual—American Association of Blood Banks.

